# Monitoring Injected CO_2_ Using Earthquake Waves Measured by Downhole Fibre-Optic Sensors: CO2CRC Otway Stage 3 Case Study

**DOI:** 10.3390/s22207863

**Published:** 2022-10-16

**Authors:** Pavel Shashkin, Boris Gurevich, Sinem Yavuz, Stanislav Glubokovskikh, Roman Pevzner

**Affiliations:** 1 Centre for Exploration Geophysics, Curtin University, Perth, WA 6102, Australia; 2 Lawrence Berkley National Laboratory, Berkley, CA 94720, USA

**Keywords:** CCUS, monitoring, fibre-optic, distributed acoustic sensing, earthquake, passive seismic

## Abstract

Monitoring changes of formation properties along the well bore associated with the presence of carbon dioxide can be important for both tracking the plume inside of the primary containment and detecting leakage into the zone located above the reservoir. This can be achieved with time lapse wireline logging, but this approach requires well intervention and is not always possible. If the well is permanently instrumented with an optical fibre, it can be used as a distributed seismic receiver array to detect gas behind the casing by monitoring changes in amplitude of the seismic waves generated by active or passive seismic sources. Previous research showed the efficacy of this technique using continuous seismic sources. The Stage 3 Otway Project presented an opportunity to test this technique using passive seismic recording, as downhole fibre-optic arrays recorded numerous regional earthquakes over the period of nearly 2 years before, during, and after CO_2_ injection. Analysis of P-wave amplitudes extracted from these downhole gathers shows a consistent amplitude anomaly at the injection level, visible in all events that occurred after the start of injection. This indicates that the anomaly is caused by changes in elastic properties in the reservoir caused by CO_2_ saturation. However, extracted amplitudes show significant variability between earthquakes even without subsurface changes; thus, multiple events are required to distinguish the time-lapse anomaly from time-lapse noise. Ubiquity of these events even in a tectonically quiet region (such as Australia) makes this technique a viable and cost-effective option for downhole monitoring.

## 1. Introduction

Time-lapse or 4D reflection seismic monitoring is an important technology for monitoring CO_2_ geosequestration, oil and gas production, and geothermal projects. Some of these projects, e.g., monitoring CO_2_ storage, require seismic surveys to be acquired as frequently as possible to ensure timely detection of unwanted subsurface changes such as CO_2_ leakage from the target reservoir. However, performing 3D seismic surveys every few months may be prohibitively expensive and disruptive for other land users. Both of these factors can be partly mitigated by the use of permanent receivers such as distributed acoustic sensors or DAS [1,2,3,4,5] and/or sources [6]. Recently, Pevzner, et al. [7] demonstrated how the multi-well offset vertical seismic profiling (VSP) using downhole DAS receivers and permanent seismic sources can continuously track a small CO_2_ plume, producing an image of the subsurface as frequently as every 2 days. 

In addition to signals from controlled sources, permanent receivers such as DAS record an abundance of seismic energy from various natural sources and human activities (earthquakes, mine blasts, ocean waves, machinery, and vehicular traffic) [8,9,10]. Harvesting some of this energy can potentially complement or even replace for certain tasks active seismic sources, reducing the cost of monitoring even further. One source of ambient seismic energy is seismicity induced by the fluid injection or extraction [11]. While induced seismicity offers valuable insights into geomechanical behaviour of the subsurface, it often lacks spatial spread required for subsurface characterisation [12,13,14,15].

Another powerful technology for subsurface characterisation is ambient noise tomography (ANT) [16,17,18,19,20]. ANT uses random seismic energy, which is in abundance in most settings. However, ANT is mainly based on analysis of surface waves; thus, its vertical resolution is much coarser than in reflection seismology.

Recent studies suggest that subsurface changes can be also monitored by tracking changes in seismic amplitudes based on a simple principle that formation softening causes an increase of displacement or strain [21,22,23]. Pevzner, et al. [23] showed that, for a P-wave propagating along the well, the strain amplitude measured by downhole DAS is proportional to ${\left(\rho V_{P}^{3}\right)}^{-1/2}$, where ρ is the formation density and $V_{P}$ is the P-wave velocity, and they demonstrated the validity of this relationship through a comparison between DAS and well-log data. Kazei, et al. [24] expanded the theory for high-contrast medium using synthetic active VSP data. Pevzner, et al. [25] demonstrated that this approach can detect the presence of CO_2_ in a monitoring well by using active seismic source data from the CO2CRC Otway Stage 3 experiment, where 15 kt of supercritical CO_2_-rich mixture was injected into a saline aquifer 1.5 km beneath the surface. During the experiment, the DAS array also recorded many regional earthquakes. This provides an opportunity to test the performance of the technique in a purely passive mode by using body waves from these earthquakes.

## 2. Theory

In the method of Pevzner, et al. [23], the use of amplitudes of earthquake waves to monitor temporal variations of elastic properties assumes that the earthquake sources are sufficiently remote so that the P-wave arrives at the well as a refracted or a diving wave that can be approximately considered as a plane wave travelling upwards along the borehole (which is assumed vertical) [26]. The method is based on the fact that the strain amplitude measured by DAS at a given point in space and time depends on elastic properties at that point (the softer the formation, the larger the strain). This behaviour can be presented in the form

(1) $$\epsilon =kY^{-1},$$

where

(2) $$Y={\left(\rho V_{P}^{3}\right)}^{1/2},$$

has been called DAS impedance [22]; k is a proportionality constant, which depends on the wave’s energy flux (which is assumed approximately constant along the well) and varies vastly between events depending on their energy and epicentral distance but is constant along the well length. Note that Equation (2) is specific for strain (or strain rate) measurements. For displacement measurements, Y would have to be replaced by acoustic impedance $\rho V_{P}$. This analogy explains the term DAS impedance [22]. 

In active seismic, sources are approximately stable over time; thus, measured strain (or strain rate) over time is directly proportional to the changes in the DAS impedance Y [25]. However, this approach is not directly applicable to earthquakes because strain in an earthquake wave depends first and foremost on the size of the earthquake, which varies between earthquakes and is a priori unknown. However, for most of the length of the well, the rock properties can be assumed constant over time and equal to the values obtained from (pre-injection) well logs. Since, for each earthquake, k is constant along the well, Equation (1) is valid at each point along the well (except for the interval(s) undergoing temporal property variations) and can be treated as a system of many equations with one unknown, k. Solving this system of equations using least squares provides a robust value of k for each earthquake. Then, for each earthquake, the DAS impedance can be obtained at every point along the well (including intervals with property variations) by solving Equation (1) for Y at each depth point. This gives the profile of DAS impedance along the well (as a function of depth z). Time-lapse changes can then be identified by comparing evolution of these profiles over so-called slow time (time on the scale of the injection or extraction experiment). 

## 3. Experiment Design

**Figure 1:**
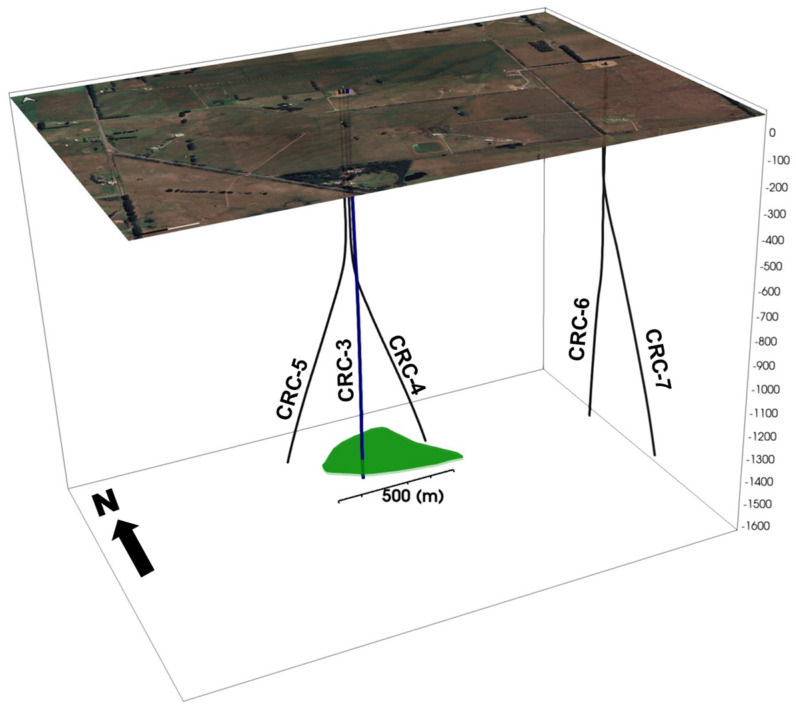
Well trajectories and CO_2_ plume position (green). Injector well CRC-3 (marked blue) is the only well crossing the plume.

The CO2CRC Otway Project Stage 3 is focused on downhole monitoring of a small-scale injection of CO_2_-rich mixture into a saline aquifer at a depth of 1.5 km, known as the Paaratte formation. For this purpose, five wells, including one injector (CRC-3, vertical) and four deviated monitoring wells (CRC-4-7), were drilled at the Otway International Test Centre in the Australian state of Victoria, in an area of approximately 1 km^2^. All wells are instrumented with fibre-optic sensing cables deployed behind the casing used for continuous DAS (and distributed temperature sensing (DTS)) recording. All the wells were drilled to a total depth of approximately 100 m below the injection interval. Well trajectories and the simplified interpreted CO_2_ plume contour are shown on Figure 1.

DAS data have been acquired continuously in all of these wells and used for active [7] and passive monitoring, commencing 22 April 2020. The event detection and analysis use data recorded until the end of December 2021, which covers 7 months of pre-injection data, the entire CO_2_ injection period (4 months), and at least 8 months after the end of injection, apart for a few operational gaps (less than 3.5% of the overall observation period).

All of the fibre-optic cables are connected to three DAS interrogators, having either a pair of wells or a well and a 1 km long trenched helically wound cable. 

## 4. Data Analysis Workflow

### 4.1. Event Detection

Composition of the ambient seismic energy recorded by DAS in the downhole environment was studied previously in a number of Australian wells instrumented with fibre-optic cables, including CRC-2 and CRC-3 [10,27], a training well at the Curtin University campus [8], and South West Hub [9] in Western Australia. These studies show strong surface waves caused by the interaction between ocean and land (oceanic microseisms), and surface and body waves generated by earthquakes, mine site blasts, and other events related to human activity. In all these events, energy propagates through the earth as seismic waves. In addition, some DAS records show events originating within the well and propagating along the elements of the well design, such as tubing, cables, or uncemented casing string sections. All these components have different spectral characteristics and travel time curves. 

In particular, oceanic microseisms are located within the 0.1–1 Hz frequency band and mostly propagate as surface waves. Distant large earthquakes have a significant amount of energy in frequencies below 1–2 Hz. These events are easily detectable but not particularly suitable for amplitude analysis as their direct body-wave arrivals are usually severely scattered over the long raypath. On the other hand, the event must be sufficiently distant from the observation site to ensure that the waves are refracted sufficiently deep, emerge at steep angles, and propagate along the wellbore as plane waves. For most of the shallow crustal earthquakes occurring few tens of kilometres away, this condition is likely to be automatically fulfilled. From observations of many DAS seismograms, it appears that refracted waves from regional events with epicentral distances of 50 to 200 km are most suitable for amplitude analysis. 

The first stage in the analysis is detection of these events. The detection workflow comprised several steps:

1. Detect all events around the time corresponding to the injection using a modified short time average over long time average (STA/LTA) algorithm [28].
2. Manually sort these events and pick travel time curves for P and S waves for several target events.
3. Scan the entire dataset using a semblance-based algorithm similar to the one applied to the pilot data acquired in CRC-3 ahead of drilling the most recent Otway wells (2018–2019) [27] using one pair of travel time curves per well representing regional earthquakes as obtained in the previous step.

For the STA/LTA scan, we used the following parameters:

- Well: CRC-3 and CRC-4, all traces below 200 m,
- Date start: 01/12/2020,
- Date end: 18/2/2021 (CRC-4); 16/03/2021 (CRC-3),
- STA window: 64 ms,
- LTA window: 800 ms,
- STA/LTA detection threshold: 60 (for a sum of STA/LTA ratios for all channels, ~300).

Prior to calculation of STA/LTA values, the data are filtered with bandpass Ormsby filter (2–3–100–240 Hz) to exclude the influence of the oceanic microseisms and large distant earthquakes. 

This procedure produces several hundred events, with a large proportion of false alarms. Signals were then manually inspected, and some of the events were used to build the travel time curve library for the semblance-based detector.

To detect the events using the semblance-based algorithm, we selected two boreholes, one from each drill pad, namely, CRC-4 and CRC-7.

The parameters were selected as follows:

- Semblance calculation time window: 30 ms,
- Combined weighted semblance (70% for P-wave and 30% for S-wave) threshold: 0.3,
- Minimal event separation: 30 s,
- Bandpass filter: Ormsby, 10–20–100–240 Hz.

All detected signals were manually verified and only events having similarity to natural regional earthquakes were added to the catalogue. In total, this workflow detected 174 events originated away from the Otway site. Out of these 174 events, 23 are present in the catalogue provided by Geoscience Australia [29] with magnitudes ranging from ~2 to 5.9 and distances from the site from 80 to 645 km. As discussed earlier, large distant events are not useful for the study and, thus, were excluded from further analysis.

**Figure 2:**
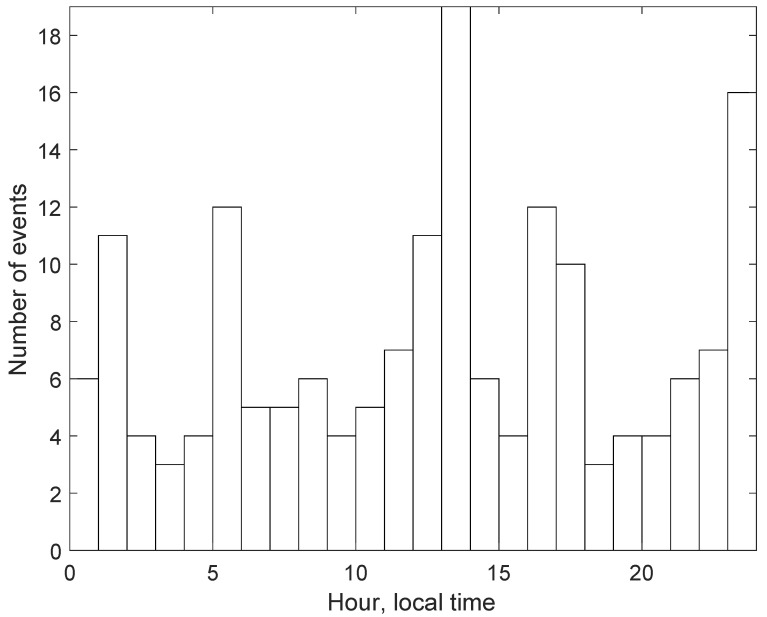
Distribution of seismic events over the time of the day.

The 174 earthquakes can either be natural seismic events or originate from human activity, such as mine site blasting. Unlike the situation close to Perth (Western Australia), where most events are attributed to mine blasting [8,9], in Victoria, a significant proportion of the events appear to be natural. This conclusion stems from the analysis of the distribution of these events through the time of the day as shown in Figure 2. Many of the events are detected at night time, when open-cut mine blasts are unlikely [30]. Furthermore, there appear to be no underground mines in the area. 

### 4.2. Event Location 

The DAS-instrumented wells employed to detect the events are located within an area of ~1 km^2^. While having such a densely instrumented site is ideal for location of local induced seismicity, this is far from optimal for regional seismic events. The system is too small to get the locations reliably but still gives some approximate estimates.

To this end, we need to estimate the distance and azimuth to the event. Approximate epicentral distance can be obtained from the difference between P and S wave arrival times (where both arrivals are identified). The distance D can be calculated as follows:

(3) $$D=\left(T_{S}-T_{P}\right)C,$$

where $T_{S}-T_{P}$ is the time separation between the arrivals, and C is a calibration constant. Assuming that the energy from an earthquake propagates as a head wave and the constant velocities of P ($V_{P}$) and S ($V_{S}$) waves, $C=V_{P}V_{S}/\left(V_{P}-V_{S}\right)$. However, these assumptions are too crude as velocities of refracted waves themselves depend on the epicentral distance (more distant events refract deeper and, thus, propagate with a higher velocity). A more accurate estimate of C can be obtained from a number of events with locations known from the catalogue. The list of the events used for calibration is provided in Table 1.

**Figure 3:**
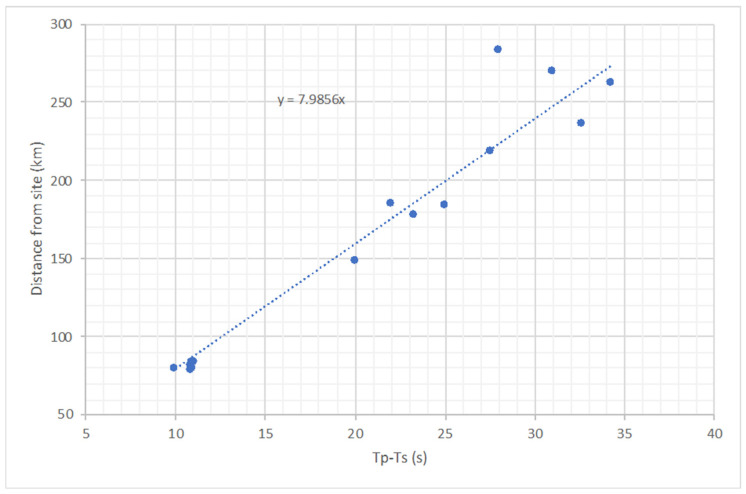
Distance to event epicentre versus the difference between P and S wave arrival times.

Figure 3 shows the distance from the site plotted against $T_{P}-T_{S}$ in. The calibration constant C=7.99. This is slightly larger, but comparable to the value for the Perth area, where C=7.5 [8].

Out of all 174 events, 119 had identifiable P and S waves. Using data from multiple wells corresponding to the same vertical depth, it is possible to search for azimuth and apparent horizontal velocity, which would minimise the misfit between the observed and theoretical arrival times for the P waves. This implies that the wave is a locally plane wave, and the velocity in the vicinity of the site is laterally uniform.

**Figure 4:**
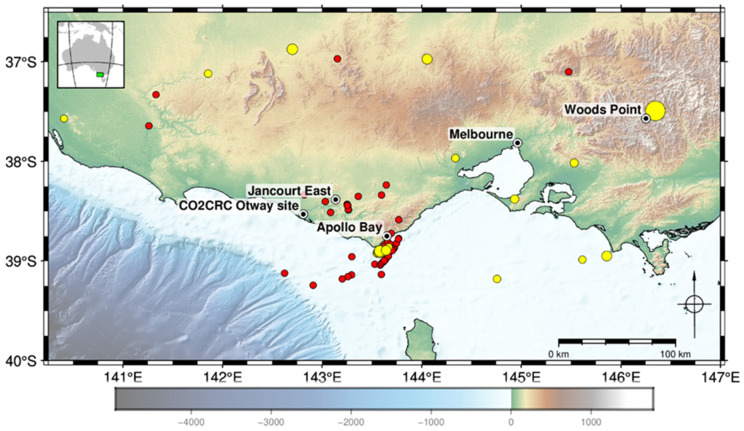
Located earthquake epicentres (red) and event epicentres from catalogue (yellow).

Azimuths to the epicentre were obtained by picking and analysing first breaks of P wave arrivals. We used the closest receiver to 1300 m TVD for each well and only those events which have the valid picks from at least three wells. The result of the procedure is shown in Figure 4.

In general, more earthquakes were detected closer to the site. This demonstrates the decay of sensitivity with the distance from the site. The distance to the epicentre of the furthermost event we were able to locate was ~284 km.

Surprisingly, a large portion of the events originated from two distinct clusters. One was located offshore in the vicinity of Cape Otway and the town of Apollo Bay (at least 31 events). The other was near the Jancourt East (11 events). Note that the Apollo Bay cluster was active over the entire observation period. This clustering may have important practical significance for reservoir monitoring using earthquakes. More or less permanent location of the epicentres can improve repeatability of the observations and can perhaps be considered in the design of receiver arrays.

**Figure 5:**
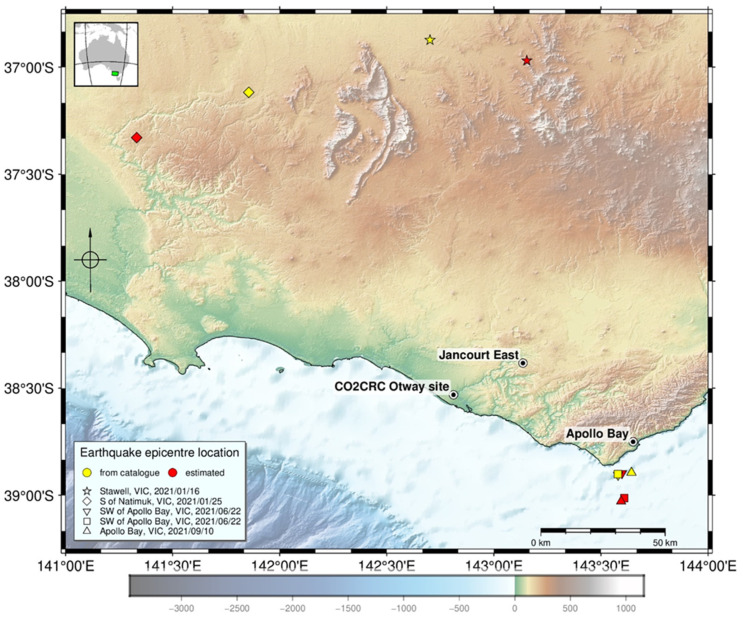
Misfit between locations of events from GA catalogue (yellow) and DAS data analysis (red).

While the accuracy of the location is not easy to estimate, it is possible to get some information about it by comparing these estimates against catalogue locations. Figure 5 shows five such events. Two events west of the Otway site are approximately 180 km away. The misfit between the locations for them is 40–50 km, which is mainly caused by errors in the azimuth, while the relative error in the distance to the epicentre is much smaller. The other three events are from the Apollo Bay cluster. Misfit in the location varies from ~2 to 14 km.

### 4.3. Amplitude Analysis

Amplitude analysis was performed on several events with almost the same *T_S_*–*T_P_* delay (most probably from the Apollo Bay Area). The data were pre-processed with a bandpass filter (2–5–20–30 Hz). The lower cut-off frequency was selected as frequencies below 2 Hz are usually dominated by ocean microseisms (ocean surface waves converted into surface or body waves in the crust; see, e.g., Glubokovskikh, et al. [10]). Frequencies higher than 30 Hz were filtered out as usual inspection showed no coherent signal from the selected events above that frequency (due to seismic attenuation).

Amplitudes were estimated as the root-mean-square values in the 1 s time window starting 200 ms before the strongest arrival for each trace corresponding to each depth in the well. Next, the values from different events were normalised to a common level, using the algorithm for decomposition of amplitude scalars for surface-consistent amplitude correction in reflection seismology (treating earthquakes as sources and DAS channels as receivers). After decomposition, only the source part of coefficients was applied to the amplitudes. The results are presented in the next section.

## 5. CO_2_ Plume Detection Results

**Figure 6:**
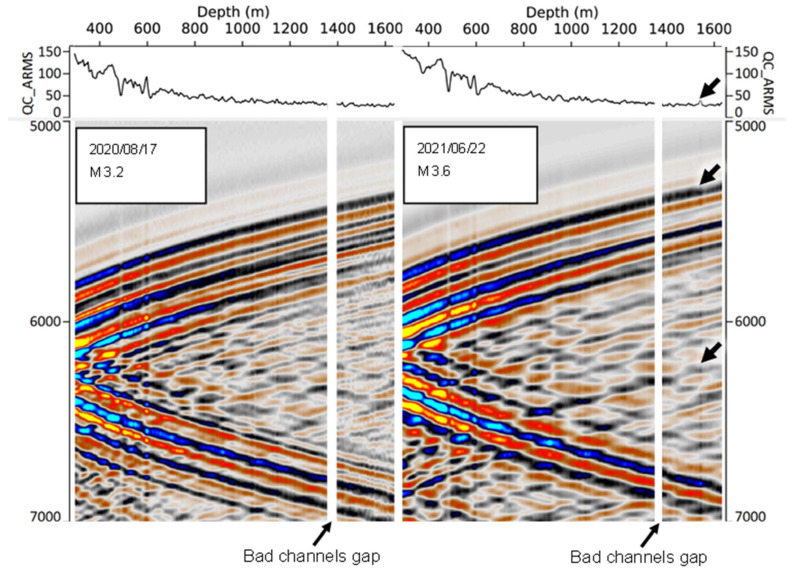
Examples of CRC-3 DAS gathers for earthquakes before and after CO_2_ injection. Thick and thin arrows indicate an amplitude peak in injection interval and bad channel gap, respectively.

Figure 6 shows the downhole DAS gathers of two earthquakes recorded in CRC-3: before (left) and after (right) the start of injection, with epicentres in close proximity of one another (near Apollo Bay, about 90 km east–southeast from the Otway site). The top half of each gather corresponds to the upgoing P-wave, and the bottom half corresponds to downgoing P-wave (created as a result of reflection of the upgoing wave from the free surface). The two gathers look similar, but the right gather shows a pronounced amplitude peak at a depth of around 1600 m, right at the level of the injection interval, which likely corresponds to the reduced DAS impedance of reservoir rocks after the CO_2_ injection. The gap at the 1355–1380 m depth interval corresponds to DAS channels in CRC-3 that were faulty for the duration of the acquisition. This gap is present in all CRC-3 DAS seismograms and amplitude profiles shown in this section. 

**Figure 7:**
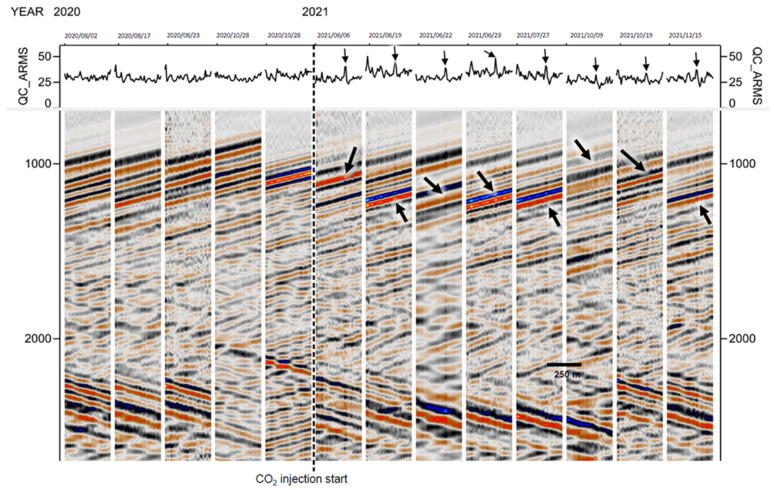
A series of CRC-3 DAS gathers in the depth interval 1380–1630 m for a selection of earthquakes before and after CO_2_ injection. Arrows indicate an amplitude peak in injection interval.

Figure 7 shows a series of downhole CRC-3 DAS gathers in a depth interval (1380–1630 m) for a number of events recorded in 2020 and 2021 (slow time increasing from left to right). The five 2020 events were all recorded before the start of the injection, while all the 2021 events were after the end of injection. The gathers represent earthquakes with a large range of sizes. Solid lines above each event show a normalised amplitude calculated as described in Section 4.3. All the 2021 events show a distinct amplitude peak at the injection interval. This amplitude peak manifests itself as a vertical strip of elevated amplitude cutting across all the arrivals included in the gather (both upgoing and downgoing arrivals reflected from the surface). This amplitude anomaly was not observed in any of the 2020 gathers and is clearly associated with a reduction in DAS impedance of rocks due to CO_2_ saturation. The strength and clarity of the amplitude anomaly varied between events, probably owning to variations of the signal-to-noise ratio, their angle to the vertical, and lateral heterogeneity of the subsurface.

**Figure 8:**
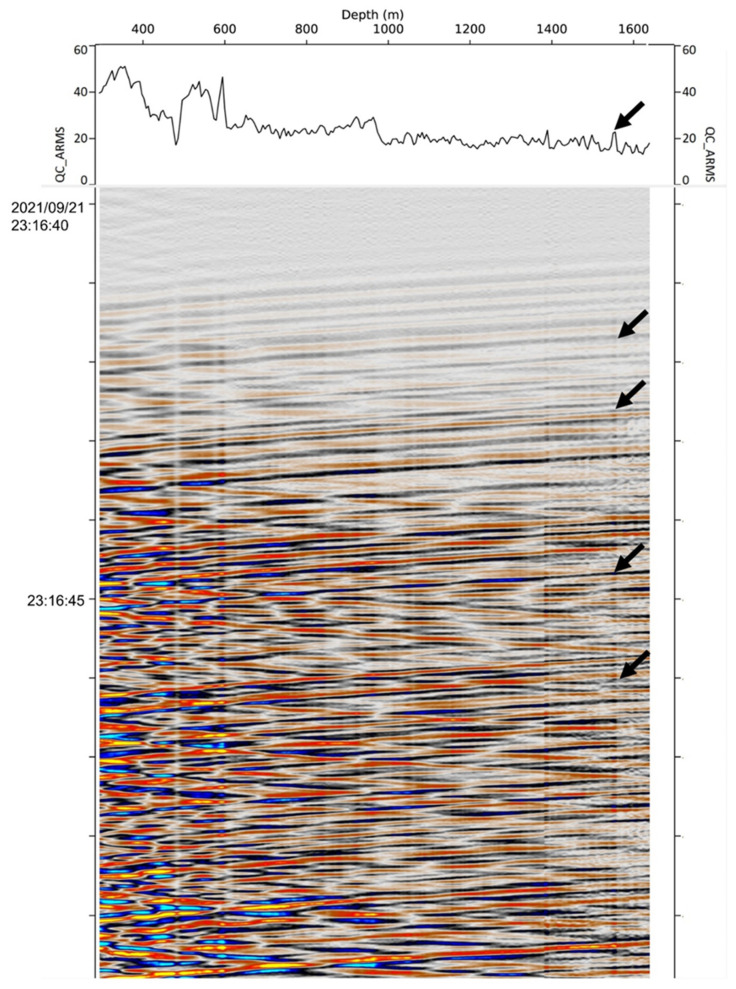
CRC-3 DAS gather of the M5.9 Mansfield Earthquake (21 September 2021). Graph above the gather shows RMS amplitude in a 1 s window around the first P-wave arrival. Arrows indicate the CO_2_ injection interval.

Figure 8 shows the CRC-3 DAS gather for the M5.9 Woods Point earthquake (22 September 2021), the largest earthquake to hit the State of Victoria in 50 years, which was strongly felt across metropolitan Melbourne and, to a lesser extent, throughout south-eastern Australia. This gather also shows a clear strip of elevated amplitudes at the injection interval.

**Figure 9:**
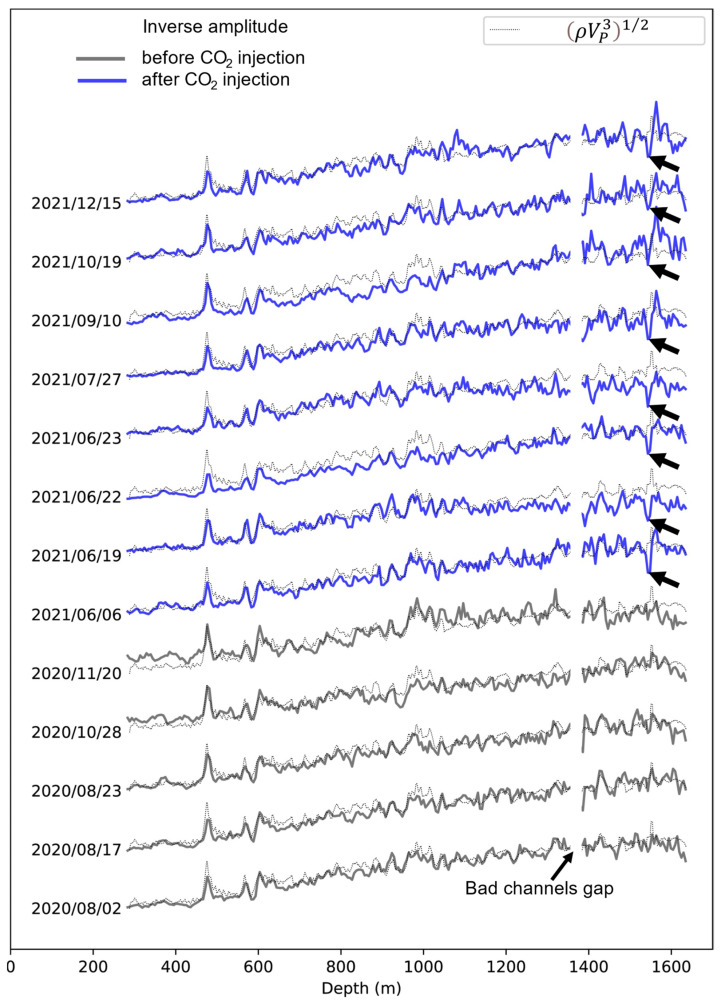
Inverse RMS amplitudes extracted from a series of CRC-3 DAS gathers of earthquakes before (black solid lines) and after (blue solid lines), plotted over DAS impedance (dotted lines) calculated from the CRC-3 well logs. Arrows indicate the amplitude an anomaly in the injection interval.

The inverse of the extracted amplitude for each of the events shown in Figure 7 is plotted in Figure 9 in comparison with the DAS impedance computed from well log data (dotted black lines). This plot shows a reasonable overall correlation of the DAS impedance (extracted from seismic amplitudes) with that calculated from sonic and density log data, although the quality of this correlation varies between events. As was seen in the gathers, all the 2021 events (blue solid lines) show a distinct trough of the DAS impedance at a depth of about 1550 m. No such trough was observed in any pre-injection (2020) events (solid black lines). These observations can be clearly interpreted as an amplitude anomaly caused by the presence of injected CO_2_. The strength (and shape) of the amplitude trough varied between events but is broadly consistent with the results obtained from active seismic analysis and forward modelling [25]. More specifically, the data presented in Figure 9 show an average decrease of the DAS impedance by about 25%, which is similar but slightly larger than with active sources (~22%) [25]; this difference is likely within the uncertainty of the method. However, the method based on earthquake waves has a coarser vertical resolution, probably due to variability of the earthquake wave signals and raypaths in deep crust. 

Note that CO_2_ injection causes not only changes in the reservoir properties but also changes of the borehole environment such as temperature, which can also affect the DAS response [31]. However, these changes are spread over a large depth range, while changes in the DAS response in our study are limited to the reservoir interval. Furthermore, temperature effects are limited to the time of injection, and should not affect the DAS response after the end of injection. 

## 6. Discussion

We demonstrated that injected CO_2_ can be detected though amplitude analysis of regional earthquake records on permanently installed downhole DAS receivers. We used the data from the injector well as it was the only well which had a CO_2_ accumulation around it at the target reservoir level. However, the value of the approach is in the ability to detect the changes in the elastic properties anywhere along the wellbore. This can be especially important for tasks such as monitoring of the unwanted migration into the so-called “above zone”, part of the subsurface located above the primary containment section. Whereas for the Otway case, the approach used natural regional earthquakes, other passive sources of body waves can be used. These include mine site blasts, which would produce similar wavefield to relatively low-magnitude earthquakes [8]. Surface sources, such as operating machinery, also generate a significant amount of body waves, which can be used in a similar way; however, in those cases, the approach needs to be modified to take into account the difference in amplitude variations caused by different divergence of the wavefront and the angle of incidence. Note that, for the earthquakes, we do not perform precision picking of travel time curves, which can probably be performed for local surface sources. 

Detection of the amplitude anomaly associated with the change in elastic properties using plane P waves is likely to be extended to the analysis of shear wave amplitudes. However, this is likely to be more complicated as those waves come after the first arrivals and interfere with other components of the wavefield. At this stage, we have not explored the possibility to separate P and S waves using some 2D filtering techniques. It is also possible that a waveform inversion technique could be developed instead of the separation. We speculate that the inversion technique can also be designed to analyse both amplitude changes and variations in travel time caused by the changes in body wave velocities.

It may also be possible to extend the technique to very-low-frequency sources of energy, such as teleseismic events. Multiple detectable events occur every day; thus, the reliability of this source is very high. Another advantage of such events is an easy separation from other noise sources in frequency domain, such as all surface events and even oceanic microseisms, which have most of the energy concentrated between 0.1 and 1 Hz. Oceanic microseisms propagate along the continental crust as Rayleigh waves with several modes [10], which also points to a possibility to use those for monitoring applications provided that an appropriate inversion technique is developed [32]. 

Like all other methods involving DAS, the proposed technique requires stability of the DAS system over monitoring time. This stability is best analysed using controlled seismic sources rather than earthquakes. The Otway project is ideal for such a test, as it operates nine permanent sources of seismic energy known as surface orbital vibrators (SOVs). Analysis of SOV/DAS data over 2 years confirms the stability of this system [7,25]. Longer-term stability is yet to be explored.

In addition to elastic properties, there are other factors which might trigger time-lapse amplitude changes. One of these is coupling of the fibre-optic receivers to the formation. Sidenko, et al. [9] reported the effect of poor cement quality in a section of a well on the DAS amplitude. This suggest that the same technique can also be employed for well integrity monitoring.

## 7. Conclusions

Nearly 2 years of passive seismic recording at the multi-well array of downhole receivers at the Otway International Test Centre revealed numerous regional earthquakes (about 10 times the number reported in catalogues). Most of this regional seismicity originated from several clusters. This ubiquity of the events (even in a tectonically quiet region) and their spatial clustering allowed the use of their P-wave amplitudes for time-lapse monitoring of formation stiffness, thus validating the method proposed in the previous studies and confirming its applicability for CO_2_ monitoring.

Analysis of P-wave amplitudes extracted from downhole DAS gathers of the earthquakes recorded over nearly 2 years of observations showed a consistent-amplitude anomaly at the injection level, visible in all events that occurred after the start of CO_2_ injection. This indicates that the anomaly was caused by a reduction in the elastic properties (DAS impedance) in the reservoir caused by CO_2_ saturation. However, due to variability of the extracted amplitudes between earthquakes even without an injection, multiple events are required to distinguish the time-lapse anomaly from time-lapse noise.

## Figures and Tables

**Table 1:** Events used for calibration.

Travel Time for P Wave (s)	Difference between P and S Wave Arrival Times (s)	Distance from Site (km)	Epicentre Location
14.0	10.0	79.23	Offshore SW of Apollo Bay, VIC, plume visible
14.5	10.9	78.35	Apollo Bay, VIC
13.8	10.9	81.92	Apollo Bay, VIC (high SNR)
14.0	11.0	78.86	Offshore SW of Apollo Bay, VIC
13.2	11.0	83.24	Apollo Bay, VIC
13.5	11.1	83.65	Apollo Bay, VIC
24.0	20.0	147.78	N of Geelong, VIC
28.5	22.0	184.29	Stawell, VIC
26.3	23.3	177.69	S of Natimuk, VIC
28.9	25.0	183.94	Bass Strait
31.5	27.6	218.31	Coastal King Island, Offshore TAS
40.7	28.0	282.57	Offshore SW of Beachport, SA
40.6	31.0	269.02	Offshore Sandy Point, VIC
33.8	32.7	235.47	Millicent, SA
38.9	34.3	261.62	Wedderburn, VIC

## Data Availability

Restrictions apply to the availability of these data. Data were obtained from Stage 3 of the CO2CRC Otway project and are available from the authors with the permission of CO2CRC Ltd.
